# Antibacterial Effect of Miswak herbal toothpaste Compared to Fluoride Toothpaste in High Caries Risk Patients: Randomized Clinical Trial

**DOI:** 10.4317/jced.60332

**Published:** 2023-07-01

**Authors:** Omar Shaalan, Aiah El-Rashidy

**Affiliations:** 1 Lecturer, Department of Conservative Dentistry, Faculty of Dentistry, Cairo University, Cairo, Egypt; 2 Lecturer, Department of Biomaterials, Faculty of Dentistry, Cairo University, Cairo, Egypt

## Abstract

**Background:**

Modern toothbrushes origin can be traced to plant-derived chewing sticks, which were documented to be used Babylonians as early as 3500 BC. Chewing sticks are routinely used for cleaning teeth in Asia, Africa, South America, and the Middle East. The aim of the current study was to evaluate the antibacterial effect of miswak herbal toothpaste compared to fluoride toothpaste using a normal toothbrush, against Streptococcus mutans in high caries risk patients.

**Material and Methods:**

A total of 32 participants were recruited to the current clinical trial using convenience sampling randomly either to miswak or fluoride toothpastes groups (n=16). The bacterial count represented as colony-forming units per milliliter (CFU/ml) was assessed at baseline and after 1 week, 1 month and 3 months. Moreover, the ion release for silicone, calcium, phosphorus, and fluoride from both toothpastes was analyzed in addition to the pH of both toothpastes and their supernatants. Intergroup comparison was performed using independent t test, while intragroup comparison was performed using repeated measures ANOVA followed by tukey post-hoc test and paired t test when appropriate.

**Results:**

There was no statistically significant difference between both toothpastes for the S. mutans count within each follow up period, however the bacterial count significantly decreased over time in both groups. Signal Fluoride toothpaste exhibited statistically significant higher ion release when compared to the Dabur Miswak toothpaste. There was no statistically significant difference between either toothpastes regarding pH (*P* = 0.5368), while comparison between supernatants of toothpastes have shown statistically significant difference (*P* = 0.0194), with a higher pH in miswak toothpaste.

**Conclusions:**

Miswak herbal toothpaste possesses a potent antibacterial effect, yet its remineralization potential is questionable due to its inferior ion release that will affect the ion substantivity in saliva, which is an important factor in remineralization.

** Key words:**High caries risk, Miswak, Fluoride, Antibacterial, Streptococcus mutans.

## Introduction

Dental caries is considered the most common infectious disease globally (1). According to the world health organization (WHO), caries of permanent teeth is the most common oral disease, where caries of permanent teeth affect about 2 billion people, and that of primary teeth affect about 520 million children (2). One of the main causative pathogens in the development of dental caries is *Streptococcus mutans* (*S. mutans*) (1,3-5) and it plays a very important role in enamel decay (4). *Lactobacilli* have also long been recognized as pathogens associated with caries (5) and plays an important role in further caries progression, especially in dentin (4).

It is well known that the best caries management procedures are those based on personalized approaches based on a consistent caries risk assessment (CRA). This led to the emergence of a relatively recent preventive approach to caries management known as “caries management by risk assessment” and abbreviated to CAMBRA® (6). CAMBRA was first published in 2007 and updated in 2019 for patients aged 6 years to adult (7), and for young children from infancy to 5 years (8). The CAMBRA CRA tool offers a risk assessment tool for these two age ranges. Clinical caries indicators are those clinically observed effects of previous and/or current caries destruction of the tooth mineral (9).

There are several clinical caries risk indicators including: clinically observed cavitation or radiographic evidence of progression into the dentin; white spot lesions onto smooth surfaces; non-cavitated demineralization into the enamel evident radiographically and existing restorations placed due to caries in the last 3 years for a new patient, or in the last year for a patient of record. “High caries risk” adult patients are usually identified by the existence of one or more of the first three disease indicators for a new patient visit, or the new appearance of any of the above disease indicators for a patient of record at a follow up visit (9).

Several protective measures are recommended for high caries risk adult patients. Tooth brushing and flossing removes dental plaque, and antiseptic mouthwashes kills some of the bacteria that help in formation of plaque, these measures aids in caries management in all patients (10). A proven management strategy for high caries risk patients includes professional fluoride varnish application every 4–6 months, brushing with a high fluoride (5,000 ppm F) toothpaste at least twice daily, and rinsing once daily for 1 week each month with a chlorhexidine gluconate mouthwash (0.12%) (6). Most of the toothpastes recommended by ADA, WHO, and FDI contains fluoride and triclosan. Excessive fluoride intake may cause some adverse effects such as skeletal fluorosis and dental fluorosis, particularly for individuals living in high-fluoride drinking water areas, and in young infants and children under age 2 years due to accidental swallowing of fluoridated toothpaste (11). In addition, the anticariogenic effectiveness of fluoride toothpastes is concentration dependent, thus, in high caries risk patients, brushing with a prescription, high fluoride (5,000 ppm F) toothpaste is recommended twice daily to lower the bacterial challenge (7).

The pH value of toothpaste is an important factor to ensure its stability and effectiveness (12). The American National Standards Institute/American Dental Association (ANSI/ADA) Standard No. 130 for Dentifrices states that the pH value of dentifrice should be less than 10.5 (13). The toothpastes must not have a low pH to avoid the dissolution of the mineral content of enamel and dentine, and to avoid the corrosion of dental prosthesis in acidic conditions (12).

*S. mutans* is a normal inhabitant of the oral cavity, it is an aciduric and acidogenic Gram-positive bacterium, which is able to metabolize various sugars and glycosides (5). *S. mutans* are highly cariogenic as they possess various virulence factors including the ability to colonize the tooth surface in large numbers in the presence of dietary sucrose, metabolize a wide array of carbohydrates, produce acid and thrive at low pH (14).

Modern toothbrushes origin can be traced to plant-derived chewing sticks, which were documented to be used Babylonians as early as 3500 BC. Chewing sticks are routinely used for cleaning teeth in Asia, Africa, South America, and the Middle East (15). ‘‘Miswak’’ is an Arabic word meaning tooth-cleaning stick, and miswak harvested from Salvadora persica (S. persica) is the most commonly used among the182 plant species suitable for preparing toothbrushing sticks. Several epidemiological studies revealed that S. persica miswak had strong anti-cariogenic effects. Chewing sticks were equally effective as conventional toothbrushes in removing oral deposits. The anticariogenic effect of miswak is mainly attributed to the mechanical brushing action, removing dental plaque and polishing the teeth (15).

However, chewing sticks as a natural toothbrush suffers from several limitations (16). The limited accessibility to the lingual surfaces of teeth is one of the main limitations, as the bristles of miswak lies in the long axis of the stick, as compared to the perpendicular orientation of the bristles in the toothbrush. In addition, miswak users were reportedly to scrub anterior teeth excessively, while ignoring posterior teeth. Improper use of miswak was also reported to be one of the etiological factors in gingival recession (16). Miswak extracts were shown to inhibit the growth of cariogenic bacteria due to their strong anti-plaque function and decreasing colonization of some streptococci strains on teeth surfaces. Miswak extract contains several antimicrobial agents such as benzyl isothiocyanate, which showed potent and fast bactericidal effect towards gram-negative bacteria and oral micro-organisms involved in periodontal disease (17). Hence, the anticariogenic properties of miswak could be attributed to its antimicrobial effects (18). Thus, the use of miswak extract-containing toothpaste may benefit from the beneficial anticariogenic effect of miswak, while avoiding the limitations of the stick.

Based on our knowledge, little information is available on the antibacterial effect of miswak extract-containing toothpaste compared to fluoride toothpaste in high caries risk patients. Thus, the current study aimed at evaluating the antibacterial effect of Dabur miswak herbal toothpaste versus Signal fluoride toothpaste, containing 1450 ppm of fluoride against *S. mutans* in high caries risk patients. In addition to the antibacterial effect, ion release of both toothpastes was analyzed for silicone, calcium, phosphorous and fluoride in order to correlate between ion release and the antibacterial effect. The null hypothesis tested that there was no difference between Dabur miswak herbal toothpaste and Signal fluoride toothpaste in minimizing *S. mutans* count in saliva of high caries risk patients.

## Material and Methods

-Trial registration and study design:

All procedures performed in the present trial were in accordance with the ethical standards of Research Ethics Committee of Faculty of Dentistry, Cairo University, (Ref. 28/9/22), informed consent was obtained from all participants. A protocol was registered in ClinicalTrials.gov ID: NCT05109299. The current study was designed as randomized clinical trial, with parallel study design, 1:1 allocation ratio and superiority framework.

-Sample size calculation:

In a previous study (19) the antibacterial effect within fluoride toothpaste group was normally distributed with standard deviation 0.458. By adopting medium cohen’s d effect size of 0.5 as a difference between fluoride and miswak toothpastes, we needed to study 14 experimental subjects and 14 control subjects to be able to reject the null hypothesis that the population means of the experimental and control groups were equal. Sample size was increased by 20% to compensate for dropouts during follow-up to be 16 per group (32). The Type I error probability associated with this test of this null hypothesis was 0.05 and type 2 error probability was 0.2 with a power of 80%.

-Eligibility criteria:

Inclusion criteria were high caries risk patients, with at least one carious tooth according to CAT caries risk assessment model, and patient compliance. Exclusion criteria were participating in another trial, using another antimicrobial agent since one month of sampling, systemic diseases or concomitant medication affecting salivary flow, parafunctional habits, and pregnancy.

**Figure 1:**
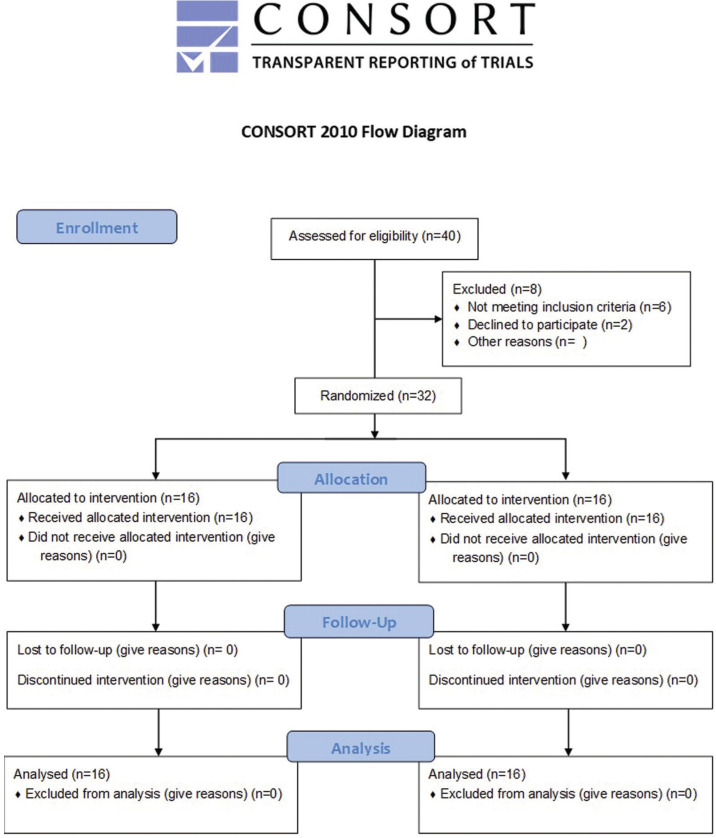
Consort flow diagram.

-Recruitment:

Participants were enrolled 1 month before the intervention from clinic of conservative dentistry, Faculty of Dentistry at Cairo University, from which eligible patients were recruited to fulfill the eligibility criteria. Procedures were explained to patients who agreed to participate in the clinical trial and informed consent was obtained from every participant prior to the research procedures. Consort flow diagram showing participants’ flow through each stage of the current randomized clinical trial (Fig. 1).

-Allocation of participants:

A total of 32 participants were recruited in the current clinical trial using convenience sampling. Simple randomization was done by generating numbers from 1:32 through random sequence generator (Randomness and Integrity Services Ltd) into two columns. The microbiologist was blinded to the material assignment.

-Interventions:

Before starting the tooth brushing procedures, a demonstration was done for all participants on a dental model using bass technique. This was followed by supervised tooth brushing to ensure adherence to the tooth brushing protocol. Each participant received the toothpaste according to the materials’ assignment and a soft manual toothbrush (UltraThin Pro Gum Care Extra, Oral B, Procter & Gamble, Schwalbach am Taunus, Germany). Participants were instructed to brush their teeth for two minutes using the bass technique, twice per day using the soft tooth brush and the assigned toothpaste (20). Toothpastes composition, manufacturers and active ingredients are listed in Table 1.

**Table 1:**
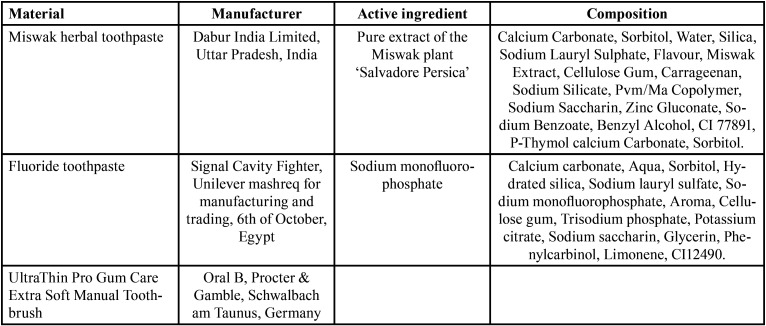
Materials used in the current study with their manufacturer, active ingredient, and composition.

-Outcome assessment:

Salivary sampling:

Salivary samples were collected at baseline before intervention, 1 week, 1 month and 3 months after intervention. Salivary samples were used to assess the *S. mutans* count before and after using the tested toothpastes. On the day of salivary sample collection, participants were asked not to drink or eat for 1 hour before sample collection. The participants were instructed to rinse with water only before saliva sample collection to prevent any contamination from food debris (21). Salivary samples were obtained between 10:00 and 11:00 am to follow the normal circadian rhythm. The patients were instructed to sit upright and let the saliva to be collected passively, swallowing was prohibited for 5 minutes, this was followed by expectoration into sterile graduated falcon collection tube through a sterile funnel (22).

Culture of *S. mutans*:

Collected salivary samples were homogenized by forceful shaking in a vortex for 30 seconds to scatter aggregates of bacteria. A calibrated micropipette was used to dilute 100 µl of saliva with 1 ml of brain heart infusion (BHI) broth to get 1:10 dilution. Dilution procedure was repeated to acquire a dilution of 1:1000000 (106) (22).

*S. mutans* was cultured on mitis salivarius-bacitracin agar (MSBA). 10 μl aliquots of were applied onto MSBA solid media using a calibrated micropipette under firm aseptic conditions. A sterile glass rod was used to produce consistent bacterial growth. The culture plate at each follow-up was incubated for 48 hours in an anaerobic atmosphere of 5–10% of carbon dioxide at 37°C using a candle jar in a precision incubator (23). After the incubation, *S. mutans* colonies appeared small, rough, raised, and adherent on the culture plate. The colonies were counted by the microbiologist as colony-forming units per milliliter (CFU/ml).

Ion release and pH measurements:

The release of silicon, calcium, phosphorus, and fluoride from both toothpastes were analyzed in triplicates (n=3). First, each toothpaste was mixed with deionized water (DIW) at a 1 mg/ml ratio, and the mixtures were stirred for 1 hour using magnetic stirrer until well dispersed. Then the mixtures were centrifuged for 30 minutes until clear supernatants were obtained. The silicon, calcium and phosphorus ions release were quantified from the supernatants using a triple-quadrupole inductive coupled plasma-mass spectrometry (ICP-MS/MS) instrument (ICP-QQQ, Agilent 8800, Agilent Technologies, Japan). Fluoride ion release was measured using fluoride specific ion electrode (ORION 9409, Thermo Fisher Scientific, MA, USA). The fluoride measurement was done through adding 3 ml of TISAB II (Total ionic strength adjustment buffer, 940909, Thermo Orion Research, Inc, Beverly MA, USA) to 3 ml of each supernatant solution, the solutions were then mixed using magnetic stirrer for one minute before placing the electrode in the mixed solutions.

The pH of both toothpastes and its supernatant were measured in triplicates (n=3) using pH meter (HANNA instruments HI 98103 PH checker tester, United States), according to the method described by Cheng *et al*. (12) with some modifications. Briefly, the pH meter was calibrated before measurements and between each set of measurements. The pH electrode tip was directly inserted into either the paste or the supernatant and left for at least 5 min to ensure stabilization of pH value measurement. Following each measurement, the electrode tip was thoroughly washed with distilled water and dried, to ensure removal of any traces of samples tested.

-Statistical analysis:

Data was analyzed using Medcalc software, version 19 for windows (MedCalc Software Ltd, Ostend, Belgium). Data was explored for normality using Kolmogrov Smirnov test and Shapiro Wilk test. Continuous data showed normal distribution and were described using mean and standard deviation. Intergroup comparison was performed using independent t test, while intragroup comparison was performed using repeated measures ANOVA followed by tukey post-hoc test and paired t test when appropriate. A *P* value less than or equal to 0.05 was considered statistically significant for intergroup comparison, while for intragroup comparison of bacterial count within each toothpaste a Bonferroni corrected *P* value was used (*P* ≤ 0.0083) and all tests were two tailed.

## Results

-Demographic data:

The present clinical trial was conducted on 32 patients that were randomly allocated to the intervention and the control arms (n=16). After 3 months all participants were assessed with 100% retention rate. There were 14 males (43.75%) and 18 females (56.25%) in the current study, there was no statistically significant difference regarding gender between groups (*P* = 0.4830). Mean age in the present study was 21.3±2.5, there was no statistically significant difference regarding age between groups (*P* = 0.405). Baseline characteristics of participants in the current study is shown in Table 2.

**Table 2:**
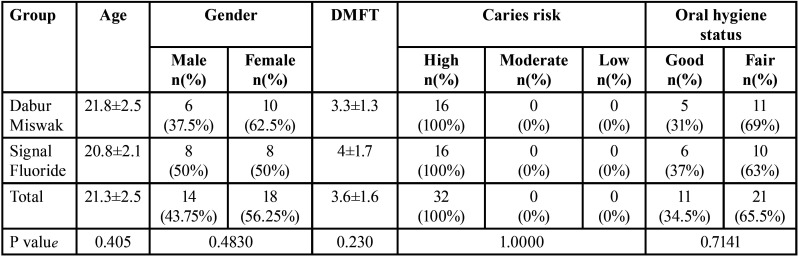
Baseline characteristics.

**Figure 2:**
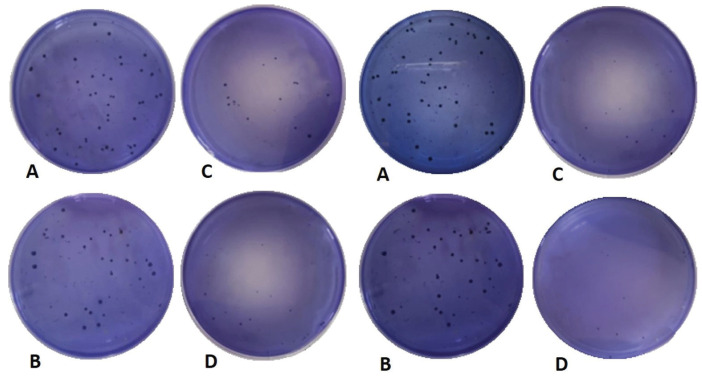
CFU/ml for Miswak toothpaste (Right) and Fluoride toothpaste (Left): A- Baseline; B- 1 week; C- 1 month; and D- 3 months.

-*Streptococcus mutans* count:

Intergroup comparison between both toothpastes have shown no statistically significant difference within follow up periods; baseline, 1 week, 1 month and 3 months (*P* = 0.7919, *P* = 0.7505, *P* = 0.5795 and *P* = 0.5490) respectively. Intragroup comparison within both toothpastes have shown statistically significant difference between different follow-up periods (*P* < 0.0001), where the bacterial count significantly decreased with time (Table 3a). A representative compiled Figure for streptococcus mutans count (CFU/ml) for both toothpastes at different follow-up periods is shown in Figure 2.

**Table 3:**
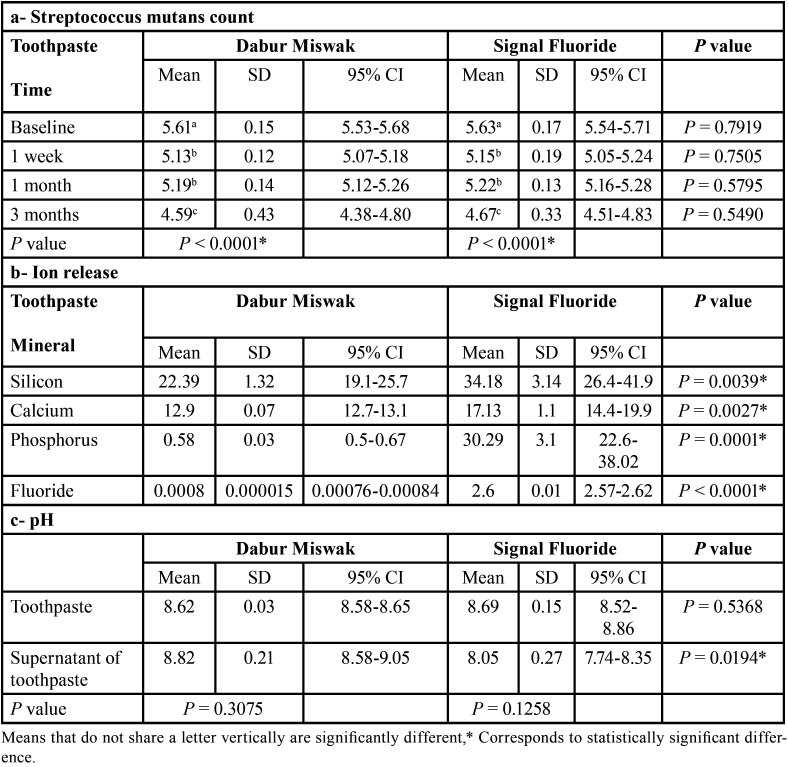
a- Mean and SD of Log (10) of CFU of streptococcus mutans count for both toothpastes after different follow-up periods; b- mean and standard deviation of ion release of silicon, calcium, phosphorus, and fluoride for both toothpastes represented as parts per million (ppm); c- mean and standard deviation of pH for miswak and fluoride toothpastes and supernatants.

-Ion release and pH measurements:

Intergroup comparison between both toothpastes showed that Signal Fluoride toothpaste released statistically significantly higher silicone, calcium, phosphorus, and fluoride ions release, as compared to the Dabur Miswak toothpaste (*P* = 0.0039, *P* = 0.0027, *P* = 0.0001 and *P* < 0.0001) respectively (Table 3b).

In the current study the mean pH of Miswak toothpaste was 8.62, while after transforming the paste into supernatant the mean pH increased to 8.82, but there was no statistically significant difference between them (*P*= 0.3075). However, for Signal Fluoride toothpaste the mean pH was 8.69, while after transforming the paste into supernatant the mean pH decreased to 8.05, also there was no statistically significant difference between them (*P* = 0.1258). Intergroup comparison between miswak and fluoride toothpastes have shown no statistically significant difference (*P* = 0.5368), while comparison between supernatants of miswak and fluoride toothpastes have shown statistically significant difference (*P* = 0.0194), with a higher pH in miswak toothpaste (Table 3c).

## Discussion

Dental caries is a multifactorial disease involving susceptible tooth structure, cariogenic microbial biofilm formed on the tooth surface, and fermentable carbohydrates, as well as environmental factors. Caries is a dynamic process which results in alternating periods of tooth demineralization and remineralization cycles, ultimately leading to mineral loss in the tooth’s hard structures, resulting in a visible cavitation (24). Hence, the efficacy of any caries preventive measures relies on the prevention of further bacterial colonization, inhibition of enamel demineralization, and enhancement of remineralization of the demineralized enamel surface.

Overexposure to dietary carbohydrates promotes the production of the extracellular polymeric substances (EPS), which establish the functional and structural integrity of bacterial biofilms. In addition to production of EPS, excess fermenTable carbohydrates increase the production of acidic metabolites and accumulation of acidogenic and aciduric microorganisms, thus initiates the transition to a pathogenic biofilm community (5). *S. mutans*, among other oral Streptococci, are the first to attach to salivary glycoproteins on tooth surfaces (acquired pellicle) (25) through their specific surface polymers such as glucan and fructan, which are derived from extracellular bacterial metabolism of dietary sucrose (26). The high cariogenic potential of *S. mutans* can be attributed to three main characteristics: its ability to synthesize large quantities of EPS, which aid in the permanent colonization of hard tooth surfaces; its ability to ferment a wide range of dietary carbohydrates into organic acids (high acidogenicity), and its ability to survive and thrive in a low pH environment (high aciduricity) (27).

In the current study, both miswak and fluoride toothpastes showed potent antibacterial effect against streptococcus mutans after 3 months. The analysis of silicon, calcium, phosphorus, and fluoride ions released from Dabur Miswak toothpaste showed statistically significantly lower release as compared to those released from Signal toothpaste. In addition, both toothpastes showed slightly alkaline pH in both the paste form and the paste supernatant (Table 3c). It is well documented that the decrease in the pH of the oral cavity results in dissolution of hydroxyapatite and demineralization occurs. While increased pH results in enhancing hydroxyapatite reprecipitation and remineralization (28-30). Most importantly, high pH was reported to favour the inhibition of bacterial growth (31), particularly for aciduric bacteria as *S. mutans* (32). Thus, most manufacturers tend to formulate toothpaste composition to adjust pH value in the oral environment.

The anticariogenic effect of fluoride can be attributed to several mechanisms: inhibition of bacterial activity in the dental plaque; reducing enamel demineralization, and enhancement of remineralization of demineralized enamel, forming more acid resistant enamel mineral, fluorapatite or a fluoridated hydroxyapatite (33,34).

The therapeutic effects of miswak on oral health have been widely reviewed (33,35-38). Several studies supported the beneficial effects of miswak as it has shown antibacterial, antifungal, antiviral, antiplaque, anticariogenic properties and promote wound healing. In addition, other studies suggested that it also has anti-inflammatory, analgesic, and antioxidant properties. S. persica miswak has a taste of spiciness, hotness or heat, which increases saliva secretion, together with the chewing effect of the stick, thus increases saliva buffering capacity. Although miswak has large amounts of fluoride, yet the anti-cariogenic impact of fluoride is questionable, due to the negligible amount of fluoride released from miswak soaked in water.

Miswak extract were shown to have strong anti-plaque properties and decrease the ability of some Streptococci to colonize teeth surfaces and prevent their attachment (18). S. persica miswak extract contains various antimicrobial agents (39), among which benzyl isothiocyanate exhibited strong and rapid bactericidal effect against gram-negative bacteria and oral pathogens involved in periodontal disease (40). Benzyl isothiocyanate were also shown to inhibit the growth of *S. mutans*, and has fungistatic action against **Candida* albicans*. In addition, miswak was reported to releases a significant amount of calcium and phosphate in water (18), in addition to fluoride and other elements, which may react with the enamel surface, thus increasing its resistance to demineralization and allow for remineralization, and may contribute to enamel resistance against carious attack (18,33,39,41,42). Silicon detected in miswak extract acts as an abrasive which may help in removing stains and plaque from tooth surfaces (15). Miswak extract was also shown to increase enamel radiodensity (42) and increase enamel microhardness (41,43) following acid challenge. The resinous content of the extract was suggested to form a layer on enamel, thus protecting against caries (15).

Prabhuswamy *et al*. in 2018 compared the antibacterial activity of different commercially available herbal toothpastes against the clinically isolated human *S. mutans* using the agar diffusion method. All the herbal toothpastes tested showed significant inhibitory effect against the *S. mutans* (44). In addition, toothpaste containing Neem showed equal anticariogenic property against *S. mutans* as compared to fluoridated toothpaste (45). Miswak toothpaste and miswak mouthwash were shown to be more effective, as compared to fluoride toothpaste, against *S. mutans* and *Lactobacilli* cariogenic bacteria, immediately after use and after 2 weeks of use (46).

In high caries risk patients, longer follow-up periods, 3-4 months, are required to re-evaluate caries risk. According to our knowledge, studies evaluating the long-term effect of miswak toothpastes on cariogenic bacteria are still limited. Thus, the current study aimed to compare the anticariogenic effect of miswak toothpaste versus fluoride toothpaste. There was no statistically significant difference between Dabur miswak herbal toothpaste and Signal fluoride toothpaste in minimizing *S. mutans* count in saliva of high caries risk patients at 1 week, 1 month and 3 months after intervention, thus the null hypothesis cannot be rejected. The bacterial count significantly decreased with time within both groups, which reflects the antimicrobial effect of miswak and fluoride toothpastes against *S. mutans*.

In the current study, although the fluoride release from the miswak toothpaste was significantly lower than that released form signal toothpaste, yet, the two toothpastes were equally effective in decreasing the bacterial count in high caries risk patients over time. Thus, the anticariogenic effect of miswak-containing toothpastes are not related to their fluoride content, in contrast to fluoride-containing ones. Thus, it may be suggested that the anticariogenic property of miswak-containing toothpastes may be attributed to the synergistic effect of its content, including benzyl isothiocyanate, and its resinous and inorganic content. Consequently, herbal toothpastes, such as miswak toothpastes, are considered a promising safe and effective anticariogenic toothpastes, especially in high caries risk patients. Limitation of the current study includes; relatively small sample size, not assessing the remineralization potential of both toothpastes on existing lesions and prevention of new carious lesions, and not measuring the actual ion release in saliva from salivary samples.

## Conclusions

Miswak toothpaste has shown similar antibacterial effect against streptococcus mutans when compared to fluoride toothpaste. However, ion release from miswak toothpaste was significantly lower than fluoride toothpaste. Therefore, miswak herbal toothpaste possesses a potent antibacterial effect, yet its remineralization potential is questionable due to its inferior ion release that will affect the ion substantivity in saliva, which is an important factor in remineralization.
